# Network impact score is an independent predictor of post-stroke cognitive impairment: A multicenter cohort study in 2341 patients with acute ischemic stroke

**DOI:** 10.1016/j.nicl.2022.103018

**Published:** 2022-04-27

**Authors:** J. Matthijs Biesbroek, Nick A. Weaver, Hugo P. Aben, Hugo J. Kuijf, Jill Abrigo, Hee-Joon Bae, Mélanie Barbay, Jonathan G. Best, Régis Bordet, Francesca M. Chappell, Christopher P.L.H. Chen, Thibaut Dondaine, Ruben S. van der Giessen, Olivier Godefroy, Bibek Gyanwali, Olivia K.L. Hamilton, Saima Hilal, Irene M.C. Huenges Wajer, Yeonwook Kang, L. Jaap Kappelle, Beom Joon Kim, Sebastian Köhler, Paul L.M. de Kort, Peter J. Koudstaal, Gregory Kuchcinski, Bonnie Y.K. Lam, Byung-Chul Lee, Keon-Joo Lee, Jae-Sung Lim, Renaud Lopes, Stephen D.J. Makin, Anne-Marie Mendyk, Vincent C.T. Mok, Mi Sun Oh, Robert J. van Oostenbrugge, Martine Roussel, Lin Shi, Julie Staals, Maria del C. Valdés-Hernández, Narayanaswamy Venketasubramanian, Frans R.J. Verhey, Joanna M. Wardlaw, David J. Werring, Xu Xin, Kyung-Ho Yu, Martine J.E. van Zandvoort, Lei Zhao, Geert Jan Biessels

**Affiliations:** a Department of Neurology and Neurosurgery, UMC Utrecht Brain Center, Utrecht, the Netherlands; b Department of Neurology, Elisabeth Tweesteden Hospital, Tilburg, the Netherlands; c Image Sciences Institute, University Medical Center Utrecht, Utrecht, the Netherlands; d Department of Imaging and Interventional Radiology, The Chinese University of Hong Kong, Hong Kong SAR, China; e Department of Neurology, Seoul National University Bundang Hospital, Seoul National University College of Medicine, Seongnam, South Korea; f Department of Neurology, Amiens University Hospital, Laboratory of Functional Neurosciences (UR UPJV 4559), Jules Verne Picardy University, 80054 Amiens Cedex, France; g Stroke Research Centre, Department of Brain Repair and Rehabilitation, UCL Queen Square Institute of Neurology, Russell Square House, 10 - 12 Russell Square, London WC1B 5EH, UK; h Université Lille, Inserm, CHU Lille, U1172 - LilNCog - Lille Neuroscience & Cognition, F-59000 Lille, France; i Neuroimaging Sciences, Centre for Clinical Brain Sciences, University of Edinburgh, Edinburgh, UK; j UK Dementia Research Institute at the University of Edinburgh, Edinburgh, UK; k Department of Pharmacology, National University of Singapore, Singapore, Singapore; l Memory, Aging and Cognition Center, National University Health System, Singapore, Singapore; m Department of Neurology, Erasmus Medical Center, Rotterdam, the Netherlands; n Saw Swee Hock School of Public Health, National University of Singapore and National University Health System, Singapore, Singapore; o Experimental Psychology, Helmholtz Institute, Utrecht University, the Netherlands; p Department of Psychology, Hallym University, Chuncheon, South Korea; q Department of Psychiatry and Neuropsychology, School for Mental Health and Neuroscience, Maastricht University, Maastricht, the Netherlands; r Division of Neurology, Department of Medicine and Therapeutics, The Chinese University of Hong Kong, Hong Kong SAR, China; s Therese Pei Fong Chow Research Centre for Prevention of Dementia, Margaret Kam Ling Cheung Research Centre for Management of Parkinsonism, Gerald Choa Neuroscience Centre, The Chinese University of Hong Kong, Hong Kong SAR, China; t Department of Neurology, Asan Medical Center, University of Ulsan College of Medicine, Seoul, South Korea; u Centre for Rural Health, University of Aberdeen, Aberdeen, UK; v Department of Neurology, Hallym University Sacred Hospital, Hallym Neurological Institute, Hallym University College of Medicine, Anyang, South Korea; w Department of Neurology, Maastricht University Medical Center, Maastricht, the Netherlands; x BrainNow Research Institute, Shenzhen, Guangdong Province, China; y Raffles Neuroscience Centre, Raffles Hospital, Singapore, Singapore

**Keywords:** Post-stroke cognitive impairment, Brain connectomics, Ischaemic stroke, Dementia, Diffusion-weighted imaging, AAL, automated anatomical labeling, CT, computed tomography, DWI, diffusion-weighted imaging, FLAIR, fluid-attenuated inversion recovery, GEE, generalized estimating equations, IQCODE, Informant Questionnaire on Cognitive Decline in the Elderly, IQR, interquartile range, MoCA, Montreal Cognitive Assessment, MNI, Montreal Neurological Institute, MRI, magnetic resonance imaging, NIHSS, national nnstitutes of health stroke scale, PSCI, post-stroke cognitive impairment, VCI, vascular cognitive impairment, WMH, white matter hyperintensities

## Abstract

**Background:**

Post-stroke cognitive impairment (PSCI) is a common consequence of stroke. Accurate prediction of PSCI risk is challenging. The recently developed network impact score, which integrates information on infarct location and size with brain network topology, may improve PSCI risk prediction.

**Aims:**

To determine if the network impact score is an independent predictor of PSCI, and of cognitive recovery or decline.

**Methods:**

We pooled data from patients with acute ischemic stroke from 12 cohorts through the Meta VCI Map consortium. PSCI was defined as impairment in ≥ 1 cognitive domain on neuropsychological examination, or abnormal Montreal Cognitive Assessment. Cognitive recovery was defined as conversion from PSCI < 3 months post-stroke to no PSCI at follow-up, and cognitive decline as conversion from no PSCI to PSCI. The network impact score was related to serial measures of PSCI using Generalized Estimating Equations (GEE) models, and to PSCI stratified according to post-stroke interval (<3, 3–12, 12–24, >24 months) and cognitive recovery or decline using logistic regression. Models were adjusted for age, sex, education, prior stroke, infarct volume, and study site.

**Results:**

We included 2341 patients with 4657 cognitive assessments. PSCI was present in 398/844 patients (47%) <3 months, 709/1640 (43%) at 3–12 months, 243/853 (28%) at 12–24 months, and 208/522 (40%) >24 months. Cognitive recovery occurred in 64/181 (35%) patients and cognitive decline in 26/287 (9%). The network impact score predicted PSCI in the univariable (OR 1.50, 95%CI 1.34–1.68) and multivariable (OR 1.27, 95%CI 1.10–1.46) GEE model, with similar ORs in the logistic regression models for specified post-stroke intervals. The network impact score was not associated with cognitive recovery or decline.

**Conclusions:**

The network impact score is an independent predictor of PSCI. As such, the network impact score may contribute to a more precise and individualized cognitive prognostication in patients with ischemic stroke. Future studies should address if multimodal prediction models, combining the network impact score with demographics, clinical characteristics and other advanced brain imaging biomarkers, will provide accurate individualized prediction of PSCI. A tool for calculating the network impact score is freely available at https://metavcimap.org/features/software-tools/lsm-viewer/.

## Highlights

• Multicenter cohort study in 2341 acute stroke patients.
• Network impact score estimates impact of infarct on brain network using information obtained from routine CT or MR imaging.
• The score was independently associated with post-stroke cognitive impairment (PSCI)
• Network impact score is a novel brain imaging biomarker for predicting PSCI.

## 1 Introduction

Post-stroke cognitive impairment (PSCI) occurs in approximately half of stroke survivors and is a leading cause of disability and reduced quality of life (Weaver et al., 2021, Barbay et al., 2019). Clinical predictors and imaging characteristics, in particular infarct location and size, have prognostic value (Weaver et al., 2021), but accurate PSCI risk prediction remains challenging. Improving PSCI risk prediction is considered an important step towards developing and implementing disease-modifying therapies in patients at risk (Biesbroek, 2022, Vinciguerra et al., 2020).

A major source of prognostic uncertainty after stroke is the marked heterogeneity in terms of underlying disease mechanisms, premorbid brain function and comorbidity, impact on brain connectomics, and brain plasticity (Biesbroek, 2022, Vinciguerra et al., 2020). As a consequence, two patients with an infarct that appears similar on routine brain imaging may have different cognitive outcomes. Promising approaches to improving prediction of PSCI focus on developing diagnostic and prognostic biomarkers that support biological definitions of disease mechanisms and quantitative, multimodal assessment of vascular brain injury and its impact on brain function (Biesbroek, 2022, Vinciguerra et al., 2020).

One specific approach to improve prediction of cognitive outcome after stroke may be to take the degree to which the infarct disrupts the brain’s network into account. Lesions in critical brain regions that have a central position in the brain network and relay information to many connected regions (i.e. network hubs) will disrupt the brain network to a larger degree than randomly located lesions (Crossley et al., 2014). Based on this concept, we recently developed the ‘network-based lesion impact score’ (henceforth called the network impact score). The network impact score primarily uses the location of the visible infarct on structural brain imaging (regular CT or MRI as used in clinical practice) to derive the impact of this lesion on the brain network, using prior knowledge (Aben et al., 2019). A lower score predicted cognitive recovery in a sample of 55 patients with PSCI in the acute stage after stroke (Aben et al., 2019). No statistically significant association with the overall presence of PSCI was found in this relatively small sample (Aben et al., 2019). A large study is needed to determine whether the network impact score is an independent predictor of cognitive outcome after ischemic stroke.

### 1.1 Aims

First, to determine if the network impact score is an independent predictor of PSCI. Second, to determine if the network impact score is an independent predictor of cognitive recovery in patients with PSCI in the subacute stage, and of cognitive decline in patients who do not have PSCI in the subacute stage. Given that the network impact score reflects the severity of global brain network disruption, we hypothesized that a higher network impact score would be associated with a higher risk of PSCI, a lower probability of cognitive recovery, and higher risk of cognitive decline.

## 2 Methods

### 2.1 Study design and study participants

We selected patients from a recently published Meta VCI Map consortium project (Weaver et al., 2021) involving 2950 patients with ischemic stroke from 12 cohorts: France (GRECogVASC (Puy et al., 2018) and STROKDEM (Bournonville et al., 2018), Hong Kong (CU-STRIDE (Zhao et al., 2018), the Netherlands (CASPER (Douven et al., 2016), CODECS (Weaver et al., 2019), PROCRAS (Aben et al., 2018), and USCOG (Biesbroek et al., 2014), Singapore (COAST (Dong et al., 2012), South Korea (Bundang VCI (Yu et al., 2013, Lim et al., 2014) and Hallym VCI (Yu et al., 2013, Lim et al., 2014), and the UK (CROMIS-2 (Wilson et al., 2018) and MSS-2 (Wardlaw et al., 2017). For all cohorts, ethical and institutional approval were obtained as required by local regulations to allow data acquisition, including informed consent, and data sharing; details for each of the 12 individual cohort are provided in the cited design papers. Inclusion criteria were CT/MRI brain imaging with a visible infarct and first cognitive assessment within 12 months after stroke. Patients with a history of stroke in addition to the index event of the study could be included. For the current study, we additionally excluded 605 patients with infarcts restricted to infratentorial regions or isolated white matter infarcts because these are not included in the brain network topology atlas used to calculate the network impact score (see section ‘network impact score calculation’ below), as well as four patients with missing data on time between stroke and cognitive assessment.

### 2.2 Cognitive assessment

Definitions for PSCI are described in detail elsewhere (Weaver et al., 2021). In short, PSCI was defined as impairment (i.e. norm-referenced <5th percentile) in ≥1 cognitive domain (attention and executive functioning, information processing speed, language, verbal memory, visuospatial perception and construction, or visuospatial memory) on neuropsychological examination and data for ≥3 domains was needed to exclude PSCI. For three cohorts only the Montreal Cognitive Assessment (MoCA) was available (CROMIS-2, CU-STRIDE, MSS-2) and PSCI was defined as norm-referenced <5th percentile performance. Three cohorts by design had only one cognitive evaluation (CODECS, GRECogVASC, USCOG), the remaining cohorts included multiple cognitive evaluations per patient. Up to six cognitive evaluations for a single patient were used. Cognitive recovery was defined as conversion from PSCI < 3 months post-stroke to no PSCI at further follow-up. Cognitive decline was defined as conversion from no PSCI < 3 months to PSCI at further follow-up.

### 2.3 Network impact score calculation

Acute infarcts were segmented on MRI or CT imaging and registered to the 1x1x1 mm resolution Montreal Neurological Institute (MNI)-152 brain template (Fonov et al., 2011) for spatial normalization. The protocol and details of this procedure are provided in the previously published Meta VCI Map consortium project from which the infarct maps were reused (Weaver et al., 2021, Biesbroek et al., 2019). The resolution of the brain images on which infarcts were segmented is provided in Table A.1. The network impact score was calculated using the infarct maps in MNI-space, the AAL atlas (Tzourio-Mazoyer et al., 2002), and the previously published brain network atlas (Aben et al., 2019) (see Figure A.1 for an illustration of the procedure). In short, the betweenness centrality of 90 cortical and subcortical grey matter regions (i.e. network nodes) was previously determined with diffusion-weighed imaging and whole-brain fiber tractography in 44 cognitively healthy adults and used to create the network atlas (Aben et al., 2019). A high betweenness centrality indicates that the node participates in a large amount of shortest paths of the brain network and that the node is considered a hub (Aben et al., 2019). For each individual patient, the network impact score is calculated as follows. First, the proportion of each of the 90 cortical and subcortical grey matter regions that is affected by the acute infarct is calculated (thus ranging from 0 to 1). Second, this proportion is multiplied by the betweenness centrality of that specific region (which is a fixed number in the network atlas). This results in an impact score for each of the 90 anatomical regions. The maximum of the 90 scores is selected as the patient’s network impact score and log transformed.

### 2.4 Statistical analyses

Generalized Estimating Equations (GEE, settings: binary logistic model with autoregressive within-subject correlation structure to take repeated measures for single subjects into account) were used to relate the network impact score to PSCI using a minimum of one and up to six repeated measurements per patient. This method allows the use of all the data per patient in a single model. Logistic regression was used to relate the network impact score to PSCI stratified according to elapsed time after stroke (<3 months, 3–12 months, 12–24 months, >24 months), and to cognitive recovery and decline. All models were adjusted for known clinical PSCI predictors based on literature (Weaver et al., 2021) (age, sex, education (categories defined by the STROKOG consortium (Lo et al., 2019), clinical history of stroke, and normalized acute infarct volume) and for study site to account for clustering. A sensitivity analysis was performed that excluded patients from the PROCRAS cohort in which the network impact score was originally developed (Aben et al., 2019). Analyses were performed with IBM SPSS Statistics for Windows, version 26.0.0.1 (IBM Corp., Armonk, N.Y., USA).

## 3 Results

**Fig. 1:**
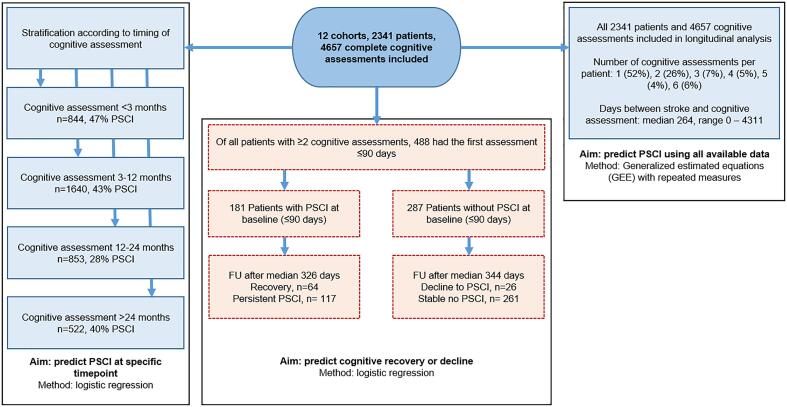
Flow chart of patient inclusion per analysis.

2341 patients met the inclusion criteria, with a total of 4657 cognitive assessments, ranging from one to six assessments per patient. Median time between stroke and the first cognitive assessment was 104 days (range 0–452). Median time between stroke and all 4657 cognitive assessments (including up to a maximum of 6 assessments per patient) was 265 days (range 0–4311). The baseline characteristics are shown in Table 1; the characteristics for each of the 12 included cohorts separately are provided in Table A.2. A flow chart of patient inclusion per analysis is shown in Fig. 1. The log transformed network impact score ranged from −3.07 to 2.46 with a higher score indicating higher impact on the brain network; boxplots per subgroup are shown in Figure A.2. An infarct prevalence map showing the distribution of infarcts is provided in Figure A.3.

**Table 1:** Baseline characteristics.

	Total sample (n = 2341)
**Demographics**	
Age in years, mean(SD)	66.7 (11.7)
Male, n(%)	1399 (59.8)
Education category n(%) - Less than high school - High school - Technical/college - University or higher	1328 (56.7) 507 (21.7) 184 (7.9) 322 (13.8)
Ethnicity, n(%)^a^ - Korean - Caucasian - Chinese - Other	1107 (47.3) 852 (36.5) 359 (15.3) 21 (0.9)

**Clinical characteristics**	
Vascular risk factors, n (%) - Hypertension ^b^ - Hyperlipidemia ^c^ - Diabetes Mellitus ^d^ - Smoking (past or present) ^e^ - Obesity ^f^ - Atrial fibrillation ^g^	1534 (67.1) 1055 (46.2) 626 (27.5) 996 (46.3) 340 (18.0) 386 (20.5)
NIHSS baseline, median(IQR) ^h^	3 (1–5)
IQCODE, median(IQR) ^i^	3.07 (3.00–3.35)
Number of cognitive assessments, n(%) − 1 − 2 − 3 − 4 − 5 − 6	1221 (52.2) 619 (26.4) 162 (6.9) 114 (4.9) 94 (4.0) 131 (5.6)
Clinical history of stroke ^j^	264 (11.3)

**Brain imaging**	
Scan sequence/modality used for infarct segmentation, n(%) - DWI - T2/FLAIR - CT - T1	1534 (65.5) 389 (16.6) 192 (8.2) 226 (9.7)
**Stroke subtypes, n (%)** - Small subcortical infarct - Cortical or large subcortical infarct, n (%)	855 (36.5) 1486 (63.5)
Normalized acute infarct volume in ml, median (IQR)	3.7 (1.2–16.1)
Imaging timing, days after stroke, median (IQR) ^k^	4 (1–8)

^a^Missing in 2 patients. ^b^Missing in 56 patients. ^c^Missing in 58 patients. ^d^Missing in 62 patients. ^e^Missing in 193 patients. ^f^Missing in 455 patients. ^g^Missing in 461 patiens. ^h^Missing in 245 patients. ^i^Missing in 791 patients. ^j^Missing in 5 patients. ^k^Missing in 1 patient. Definitions for vascular risk factors are provided elsewhere.^1^ Stroke subtypes are defined in the appendix.

### 3.1 Post-stroke cognitive impairment

The number of patients with available cognitive data varied per post-stroke interval (see Fig. 1). PSCI was present in 397/844 patients (47%) < 3 months, 709/1640 (43%) at 3–12 months, 243/853 (28%) at 12–24 months, and 208/522 (39%) > 24 months. In the GEE repeated measures model (Table 2), the network impact score independently predicted PSCI, both in the univariable model (Odds Ratio (OR) 1.50 (95%CI 1.34–1.68) per 1-point increase), and in the multivariable model (OR 1.27 (95%CI 1.10–1.46)). In the logistic regression models stratified for post-stroke time interval (Table A.3), the network impact score had comparable ORs and overlapping 95%CIs for predicting PSCI at all specified post-stroke intervals as in the GEE model, though the association was only statistically significant <3 months and at 3–12 months after stroke when correcting for age, sex, education, infarct volume, prior stroke, and study site. In the sensitivity analysis excluding patients from the PROCRAS cohort, the results were essentially unchanged (Tables A.4 and A.5).

**Table 2:** Association between network impact score and PSCI using up to six follow-up cognitive assessments per patient.

	PSCI	
Univariable model	OR per unit increase (95%CI)	p-value
Network impact score (range −3.07 to 2.46)	1.50 (1.34–1.68) ^a^	<0.001
**Multivariable model**^b^		
Network impact score (range −3.07 to 2.46)	1.27 (1.10–1.46) ^a^	<0.001
Age (per decade)	1.17 (1.08–1.27)	<0.001
Male sex (compared to female)	0.85 (0.70–1.04)	0.118
Education category (reference = less than high school)		
- High school completion	0.88 (0.67–1.15)	0.345
- Technical/college completion	0.83 (0.61–1.15)	0.266
- University or higher	1.01 (0.76–1.35)	0.936
Clinical history of stroke	1.76 (1.31–2.37)	<0.001
Total infarct volume (per 10 mL)	1.10 (1.05–1.14)	<0.001

GEE repeated measures model. The univariable model includes 4657 cognitive assessments in 2341 patients. The multivariable model includes 4651 cognitive assessments in 2336 patients (five patients were excluded in the multivariable model due to missing data on clinical history of stroke). ^a^ORs apply to each 1-point increase in the network impact score. ^b^Model also corrected for study site.

### 3.2 Cognitive recovery and decline

Cognitive recovery occurred in 64 out of 181 patients (35%) who had PSCI < 3 months post-stroke. Cognitive decline occurred in 26 out of 287 patients (9%) who did not have PSCI < 3 months post-stroke. The network impact score was not significantly associated with cognitive recovery (univariable OR 1.31, 95%CI 0.88–1.93) or decline (univariable OR 1.49, 95%CI 0.90–2.46) in both the univariable and multivariable models (Table A.6). The sensitivity analyses excluding patients from the PROCRAS cohort showed comparable results (Table A.7).

## 4 Discussion

In this multicenter study using pooled individual patient data from 2341 ischemic stroke patients from 12 cohorts, the network impact score was shown to be an independent predictor of PSCI. The network impact score did not predict change in PSCI status over time.

In the current study, the network score predicted PSCI, but the previously observed association between the network impact score and cognitive recovery in patients with PSCI (Aben et al., 2019) was not reproduced. This may indicate that the network impact score captures the impact of an infarct on cognitive functioning (and therefore the risk of PSCI), while it does not capture the potential for cognitive recovery in those patients who developed PSCI, meaning brain plasticity may be more strongly determined by other factors. Alternatively, the negative finding regarding prediction of recovery may be due to a less sensitive definition of cognitive recovery. In the current study PSCI was defined as <5th percentile norm-referenced performance on either detailed neuropsychological examination or MoCA to conform to the definitions used in the previous Meta VCI Map consortium PSCI project (Weaver et al., 2021), whereas in the original study a norm-referenced z-score < 1 cut-off was included as minor PSCI, conversion from major to minor PSCI was also considered as cognitive recovery, and only detailed neuropsychological examinations were used.

The main strengths of the study are the large sample size and geographical coverage, external validation of a previously developed score, and numerous serial cognitive assessments. Limitations include that information on pre-stroke cognitive status was not available for many patients and prior infarcts were ignored in the infarcted tissue delineation, therefore neither could be taken into account. Also, serial cognitive assessments were unavailable for 52.2% of patients, and power for predicting cognitive decline was limited because this only occurred in only 26 out of 287 cases. Furthermore, the analysis only included patients with visible acute infarcts and therefore does not apply to the 30% of patients with mild stroke who do not have visible infarcts. Finally, the median NIHSS in our study was relatively low, which means a substantial number of patients had a mild stroke. Consequently, the risk of PSCI in our study might be lower than in the general stroke population. Two of the 12 included cohorts by design excluded patients with severe stroke (STROKDEM and MSS-2) (Weaver et al., 2021). Nevertheless, the prevalence of PSCI in our study was substantial (47% within 3 months after stroke, and 43% 3–12 months after stroke) and only slightly lower than previously published estimations of PSCI risk in patients with stroke (Barbay et al., 2019).

The results indicate that the network impact score may be a useful addition to currently available brain imaging biomarkers for predicting cognitive outcome after ischemic stroke. Early identification of patients with a high risk of PSCI is helpful for patient counseling and for choosing appropriate monitoring or rehabilitation strategies. Furthermore, the identification of patients with a high risk of PSCI can be used to include these patients in intervention studies aimed at developing new treatments to improve cognitive outcome. The network impact score can be calculated during hospital admission using the location of the acute infarct on CT or MRI. We therefore envision that, with further steps of refinement and implementation, the score could be combined with other brain imaging-based biomarkers to develop an individualized prediction tool for PSCI, that may be used during the acute hospitalization period. Promising brain imaging biomarkers to include in such a prediction tool are infarct location (for example using the recently published infarct location impact score (Weaver et al., 2021), quantitative information on other vascular lesion types (e.g. burden and location of pre-existing white matter hyperintensities (WMH), lacunes and old infarcts), brain atrophy (Shi et al., 2018), and atlas-based information on white matter connectomics (given that the network impact scores includes only information on cortical and subcortical grey matter structures). All this information could be derived from routine structural MRI scans. To implement a brain imaging-based prediction tool, image processing (i.e. lesion segmentation and registration) needs to become fully automated to enable implementation in clinical settings where the required time and expertise for manual or semi-automated procedures is not available. Image processing of most lesion types are expected to become fully automated in the future given ongoing efforts in the field (e.g. automated segmentation methods for acute infarcts on DWI sequences (Zhang et al., 2018) and WMH on FLAIR images (Kuijf et al., 2019) already exist). A tool for automated calculation of the network impact score is freely available at https://metavcimap.org/features/software-tools/lsm-viewer/. Additional promising approaches to further improve PSCI prediction include assessment of co-occurring Alzheimer pathology (e.g. using cerebrospinal fluid analysis, amyloid PET imaging (Ossenkoppele et al., 2015) or blood-based measurements (Chong et al., 2021), brain perfusion (e.g. using transcranial Doppler (Vinciguerra et al., 2019) or arterial spin labeling MRI (van den Brink et al., 2021), and brain plasticity and reserve (e.g. using transcranial magnetic stimulation (Di Lazzaro et al., 2021), functional or structural connectivity measures (Santonja et al., 2021).

To conclude, the network impact score is an independent predictor of PSCI. The network impact score can be used to determine the impact of an infarct on the brain network using only routine structural MRI or CT imaging. As such, the network impact score may contribute to a more precise and individualized cognitive prognosis in patients with stroke. Additional steps needed to implement the network impact score in clinical practice are further automatization of brain image processing, incorporation of quantitative measures of other vascular lesion types and brain atrophy, and external validation.

## Author contributions

JMB and GJB: Conceptualization. NAW andHJK: image processing. JMB and HPA: network impact score calculation. NAW, IMCHW and JMB: neuropsychological data harmonization, with input from OG, OKLH, BYKL, J-SL, TD, and XX. JMB: Formal analysis. JMB: Writing – original draft. All authors: Writing – review & editing.

## Funding

This study was supported by a VIMP grant (project 7330505031) from The Netherlands Organisation for Health Research and Development (ZonMw) to JMB and GJB. The Meta VCI Map consortium is supported by Vici Grant 918.16.616 from ZonMw to GJB. The funding sources had no in role in study design, collection, analysis and interpretation of data, writing of the report, and the decision to submit the article for publication.

## Declaration of Competing Interest

The authors declare that they have no known competing financial interests or personal relationships that could have appeared to influence the work reported in this paper.

## References

[b0005] Weaver N.A., Kuijf H.J., Aben H.P., Abrigo J., Bae H.-J., Barbay M., Best J.G., Bordet R., Chappell F.M., Chen C.P.L.H., Dondaine T., van der Giessen R.S., Godefroy O., Gyanwali B., Hamilton O.K.L., Hilal S., Huenges Wajer I.M.C., Kang Y., Kappelle L.J., Kim B.J., Köhler S., de Kort P.L.M., Koudstaal P.J., Kuchcinski G., Lam B.Y.K., Lee B.-C., Lee K.-J., Lim J.-S., Lopes R., Makin S.D.J., Mendyk A.-M., Mok V.C.T., Oh M.S., van Oostenbrugge R.J., Roussel M., Shi L., Staals J., del C Valdés-Hernández M., Venketasubramanian N., Verhey F.R.J., Wardlaw J.M., Werring D.J., Xin X.u., Yu K.-H., van Zandvoort M.J.E., Zhao L., Biesbroek J.M., Biessels G.J. (2021). Strategic infarct locations for post-stroke cognitive impairment: a pooled analysis of individual patient data from 12 acute ischaemic stroke cohorts. Lancet Neurol..

[b0010] Barbay M., Diouf M., Roussel M., Godefroy O. (2019). Systematic review and meta-analysis of prevalence in post-stroke neurocognitive disorders in hospital-based studies. Dement. Geriatr. Cogn. Disord..

[b0015] Biesbroek J.M., Biessels G.J. (2022). Diagnosing vascular cognitive impairment: current challenges and future perspectives. *Int. J. Stroke*.

[b0020] Vinciguerra L., Lanza G., Puglisi V., Fisicaro F., Pennisi M., Bella R., Cantone M. (2020). Update on the neurobiology of vascular cognitive impairment: from lab to clinic. Int. J. Mol. Sci..

[b0025] Crossley N.A., Mechelli A., Scott J., Carletti F., Fox P.T., McGuire P., Bullmore E.T. (2014). The hubs of the human connectome are generally implicated in the anatomy of brain disorders. Brain.

[b0030] Aben H.P., Biessels G.J., Weaver N.A., Spikman J.M., Visser-Meily J.M.A., de Kort P.L.M., Reijmer Y.D., Jansen B.P.W. (2019). Extent to which network hubs are affected by ischemic stroke predicts cognitive recovery. Stroke.

[b0035] Puy L., Barbay M., Roussel M., Canaple S., Lamy C., Arnoux A., Leclercq C., Mas J.-L., Tasseel-Ponche S., Constans J.-M., Godefroy O. (2018). Neuroimaging determinants of Poststroke cognitive performance: the GRECogVASC study. Stroke.

[b0040] Bournonville C., Hénon H., Dondaine T., Delmaire C., Bombois S., Mendyk A.-M., Cordonnier C., Moulin S., Leclerc X., Bordet R., Lopes R. (2018). Identification of a specific functional network altered in poststroke cognitive impairment. Neurology.

[b0045] Zhao L., Biesbroek J.M., Shi L., Liu W., Kuijf H.J., Chu W.WC., Abrigo J.M., Lee R.KL., Leung T.WH., Lau A.YL., Biessels G.J., Mok V., Wong A. (2018). Strategic infarct location for post-stroke cognitive impairment: a multivariate lesion-symptom mapping study. J. Cereb. Blood Flow Metab..

[b0050] Douven E., Schievink S.H.J., Verhey F.R.J., van Oostenbrugge R.J., Aalten P., Staals J., Köhler S. (2016). The Cognition and Affect after Stroke - a Prospective Evaluation of Risks (CASPER) study: rationale and design. BMC Neurol.

[b0055] Weaver N.A., Zhao L., Biesbroek J.M., Kuijf H.J., Aben H.P., Bae H.-J., Caballero M.Á.A., Chappell F.M., Chen C.P.L.H., Dichgans M., Duering M., Georgakis M.K., Giessen R.S., Gyanwali B., Hamilton O.K.L., Hilal S., Hofe E.M., Kort P.L.M., Koudstaal P.J., Lam B.Y.K., Lim J.-S., Makin S.D.J., Mok V.C.T., Shi L., Valdés Hernández M.C., Venketasubramanian N., Wardlaw J.M., Wollenweber F.A., Wong A., Xin X., DeCarli C., Fletcher E.A., Maillard P., Barnes J., Sudre C.H., Schott J.M., Ikram M.A., Papma J.M., Steketee R.M.E., Vernooij M.W., Bordet R., Lopes R., Huang C.-W., Frayne R., McCreary C.R., Smith E.E., Backes W., Köhler S., Oostenbrugge R.J., Staals J., Verhey F., Cheng C.Y., Kalaria R.N., Werring D., Hsu J.L., Huang K.-L., Grond J., Jukema J.W., Mast R.C., Nijboer T.C.W., Yu K.-H., Schmidt R., Pirpamer L., MacIntosh B.J., Robertson A.D., Leeuw F.-E., Tuladhar A.M., Chaturvedi N., Tillin T., Brodaty H., Sachdev P., Barkhof F., Flier W.M., Kappelle L.J., Biessels G.J. (2019). The Meta VCI Map consortium for meta-analyses on strategic lesion locations for vascular cognitive impairment using lesion-symptom mapping: design and multicenter pilot study. Alzheimer’s Dement Diagnosis, Assess Dis. Monit..

[b0060] Aben H.P., Reijmer Y.D., Visser-Meily J.MA., Spikman J.M., de Bresser J., Biessels G.J., de Kort P.LM. (2018). A role for new brain magnetic resonance imaging modalities in daily clinical practice: protocol of the prediction of cognitive recovery after stroke (PROCRAS) study. JMIR Res. Protoc..

[b0065] Biesbroek J.M., van Zandvoort M.J.E., Kuijf H.J., Weaver N.A., Kappelle L.J., Vos P.C., Velthuis B.K., Biessels G.J., Postma A. (2014). The anatomy of visuospatial construction revealed by lesion-symptom mapping. Neuropsychologia.

[b0070] Dong Y.H., Venketasubramanian N., Chan B.P.L. (2012). Brief screening tests during acute admission in patients with mild stroke are predictive of vascular cognitive impairment 3–6 months after stroke. J. Neurol. Neurosurg. Psychiatry.

[b0075] Yu K.-H., Cho S.-J., Oh M.S., Jung S., Lee J.-H., Shin J.-H., Koh I.-S., Cha J.-K., Park J.-M., Bae H.-J., Kang Y., Lee B.-C. (2013). Cognitive impairment evaluated with vascular cognitive impairment harmonization standards in a multicenter prospective stroke cohort in Korea. Stroke.

[b0080] Lim J.-S., Kim N., Jang M.U., Han M.-K., Kim S.Y., Baek M.J., Jang M.S., Ban B., Kang Y., Kim D.-E., Lee J.S., Lee J., Lee B.-C., Yu K.-H., Black S.E., Bae H.-J. (2014). Cortical hubs and subcortical cholinergic pathways as neural substrates of poststroke dementia. Stroke.

[b0085] Wilson D., Ambler G., Shakeshaft C., Brown M.M., Charidimou A., Al-Shahi Salman R., Lip G.Y.H., Cohen H., Banerjee G., Houlden H., White M.J., Yousry T.A., Harkness K., Flossmann E., Smyth N., Shaw L.J., Warburton E., Muir K.W., Jäger H.R., Werring D.J., Aeron-Thomas J., Aghoram P., Amis E., Anderton P., Andole S., Anwar I., Bamford J., Banaras A., Barry A., Bellfied R., Benford A., Bhalla A., Bhargava M., Bhaskaran B., Bhupathiraju N., Birns J., Blight A., Bowring A., Brown E., Bruce D., Buck A., Bunworth K., Burger I., Burgess L., Burn M., Burssens E., Burton M., Butler N., Button D., Carpenter M., Chadha D., Chatterjee K., Choy L., Cohen D., Connell L., Cooper M., Corrigan J., Cotterill D., Courtauld G., Crawford S., Cullen C., Dani K., Daniel A., Datta P., Davis M., Day N., Doherty M., Douglas C., Dunne K., Edwards C., Eglinton C., Elmarimi A., Emsley H., England T., Epstein D., Erande R., Esisi B., Evans R., Farren P., Fitzell P., Fletcher G., Gallifent R., Gascoyne R., Giallombardo E., Gregary B., Gunathilagan G., Guyler P., Hairsine B., Haley M., Hardwick A., Hargroves D., Harrington F., Hedstrom A., Holmes C., Hussein S., Ingram T., Ispoglou S., Iveson L., Johnson V., Justin F., Kausar S., Kee K., Keeling M., Khan S., Kieliszkowska A., Kingwell H., Krishnamurthy V., Kullane S., Kumar B., Leach S., Leason S., Lopez P., Luder R., Madigan B., Maguire S., Maguire H., Mahawish K., Makawa L., Mamun M., Manawadu D., Mangion D., Manoj A., Mansoor S., Marsden T., Marsh R., Mashate S., McCormick M., McGolick C., McKee M., Mckenzie E., Meenakishundaram S., Mellor Z., Misra A., Mistri A., Mohd Nor A., Mpelembue M., Murphy P., Nallasivam A., Needle A., Nguyen V., O'Connell J., O'Mahony P., Okwera J., Orefo C., Owusu-Agyei P., Parry A., Parry-Jones A., Pasco K., Patterson C., Peixoto C., Perez J., Persad N., Porteous M., Power M., Price C., Proschel H., Punekar S., Putterill J., Randall M., Redjep O., Rehman H., Richards E., Riddell V., Roffe C., Rogers G., Rudd A., Saastamoinen K., Sajid M., Sandhu B., Schofield C., Scott J., Sekaran L., Sharma P., Sharma J., Sharpe S., Smith M., Smith A., Sprigg N., Staals J., Steele A., Storey G., Storey K., Subramonian S., Sword J., Tallon G., Tan G., Tate M., Teke J., Temple N., Thompson T., Tysoe S., Vahidassr D., van der Kwaak A., Veltkamp R., Walstow D., Watchurst C., Watson F., Waugh D., Wilkinson P., Wilson D., Wilson-Owen S., Wroath B., Wynter I., Young E. (2018). Cerebral microbleeds and intracranial haemorrhage risk in patients anticoagulated for atrial fibrillation after acute ischaemic stroke or transient ischaemic attack (CROMIS-2): a multicentre observational cohort study. Lancet Neurol..

[b0090] Wardlaw J.M., Makin S.J., Valdés Hernández M.C., Armitage P.A., Heye A.K., Chappell F.M., Muñoz‐Maniega S., Sakka E., Shuler K., Dennis M.S., Thrippleton M.J. (2017). Blood-brain barrier failure as a core mechanism in cerebral small vessel disease and dementia: evidence from a cohort study. Alzheimer’s Dement.

[b0095] Fonov V., Evans A.C., Botteron K., Almli C.R., McKinstry R.C., Collins D.L. (2011). Unbiased average age-appropriate atlases for pediatric studies. Neuroimage.

[b0100] Biesbroek J.M., Kuijf H.J., Weaver N.A., Zhao L., Duering M., Biessels G.J. (2019). Brain infarct segmentation and registration on MRI or CT for lesion-symptom mapping. J. Vis. Exp..

[b0105] Tzourio-Mazoyer N., Landeau B., Papathanassiou D., Crivello F., Etard O., Delcroix N., Mazoyer B., Joliot M. (2002). Automated anatomical labeling of activations in SPM using a macroscopic anatomical parcellation of the MNI MRI single-subject brain. Neuroimage.

[b0110] Lo J.W., Crawford J.D., Desmond D.W., Godefroy O., Jokinen H., Mahinrad S., Bae H.-J., Lim J.-S., Köhler S., Douven E., Staals J., Chen C., Xu X., Chong E.J., Akinyemi R.O., Kalaria R.N., Ogunniyi A., Barbay M., Roussel M., Lee B.-C., Srikanth V.K., Moran C., Kandiah N., Chander R.J., Sabayan B., Jukema J.W., Melkas S., Erkinjuntti T., Brodaty H., Bordet R., Bombois S., Hénon H., Lipnicki D.M., Kochan N.A., Sachdev P.S. (2019). Profile of and risk factors for poststroke cognitive impairment in diverse ethnoregional groups. Neurology.

[b0115] Shi L., Zhao L., Yeung F.K., Wong S.Y., Chan R.K.T., Tse M.F., Chan S.C., Kwong Y.C., Li K.C., Liu K., Abrigo J.M., Lau A.Y.L., Wong A., Lam B.Y.K., Leung T.W.H., Fu J., Chu W.C.W., Mok V.C.T. (2018). Mapping the contribution and strategic distribution patterns of neuroimaging features of small vessel disease in poststroke cognitive impairment. J. Neurol. Neurosurg. Psychiatry.

[b0120] Zhang R., Zhao L., Lou W., Abrigo J.M., Mok V.C.T., Chu W.C.W., Wang D., Shi L. (2018). Automatic segmentation of acute ischemic stroke from DWI using 3-D fully convolutional DenseNets. IEEE Trans. Med. Imaging.

[b0125] Kuijf H.J., Casamitjana A., Collins D.L., Dadar M., Georgiou A., Ghafoorian M., Jin D., Khademi A., Knight J., Li H., Llado X., Biesbroek J.M., Luna M., Mahmood Q., McKinley R., Mehrtash A., Ourselin S., Park B.-Y., Park H., Park S.H., Pezold S., Puybareau E., De Bresser J., Rittner L., Sudre C.H., Valverde S., Vilaplana V., Wiest R., Xu Y., Xu Z., Zeng G., Zhang J., Zheng G., Heinen R., Chen C., van der Flier W., Barkhof F., Viergever M.A., Biessels G.J., Andermatt S., Bento M., Berseth M., Belyaev M., Cardoso M.J. (2019). Standardized assessment of automatic segmentation of white matter hyperintensities and results of the WMH segmentation challenge. IEEE Trans. Med. Imaging.

[b0130] Ossenkoppele R., Jansen W.J., Rabinovici G.D., Knol D.L., van der Flier W.M., van Berckel B.N.M., Scheltens P., Visser P.J., Verfaillie S.C.J., Zwan M.D., Adriaanse S.M., Lammertsma A.A., Barkhof F., Jagust W.J., Miller B.L., Rosen H.J., Landau S.M., Villemagne V.L., Rowe C.C., Lee D.Y., Na D.L., Seo S.W., Sarazin M., Roe C.M., Sabri O., Barthel H., Koglin N., Hodges J., Leyton C.E., Vandenberghe R., van Laere K., Drzezga A., Forster S., Grimmer T., Sánchez-Juan P., Carril J.M., Mok V., Camus V., Klunk W.E., Cohen A.D., Meyer P.T., Hellwig S., Newberg A., Frederiksen K.S., Fleisher A.S., Mintun M.A., Wolk D.A., Nordberg A., Rinne J.O., Chételat G., Lleo A., Blesa R., Fortea J., Madsen K., Rodrigue K.M., Brooks D.J. (2015). Prevalence of amyloid PET positivity in dementia syndromes: a meta-analysis. JAMA.

[b0135] Chong J.R., Ashton N.J., Karikari T.K., Tanaka T., Schöll M., Zetterberg H., Blennow K., Chen C.P., Lai M.K.P. (2021). Blood-based high sensitivity measurements of beta-amyloid and phosphorylated tau as biomarkers of Alzheimer’s disease: a focused review on recent advances. J. Neurol. Neurosurg. Psychiatry.

[b0140] Vinciguerra L., Lanza G., Puglisi V., Pennisi M., Cantone M., Bramanti A., Pennisi G., Bella R., Ginsberg S.D. (2019). Transcranial Doppler ultrasound in vascular cognitive impairment-no dementia. PLoS One.

[b0145] van den Brink H., Kopczak A., Arts T., Onkenhout L., Siero J.C.W., Zwanenburg J.J.M., Duering M., Blair G.W., Doubal F.N., Stringer M.S., Thrippleton M.J., Kuijf H.J., de Luca A., Hendrikse J., Wardlaw J.M., Dichgans M., Biessels G.J. (2021). Zooming in on cerebral small vessel function in small vessel diseases with 7T MRI: rationale and design of the “ZOOM@SVDs” study. Cereb. Circ. – Cogn. Behav..

[b0150] Di Lazzaro V., Bella R., Benussi A., Bologna M., Borroni B., Capone F., Chen K.-H., Chen R., Chistyakov A.V., Classen J., Kiernan M.C., Koch G., Lanza G., Lefaucheur J.-P., Matsumoto H., Nguyen J.-P., Orth M., Pascual-Leone A., Rektorova I., Simko P., Taylor J.-P., Tremblay S., Ugawa Y., Dubbioso R., Ranieri F. (2021). Diagnostic contribution and therapeutic perspectives of transcranial magnetic stimulation in dementia. Clin. Neurophysiol..

[b0155] Santonja J., Martínez K., Román F.J., Escorial S., Quiroga M.Á., Álvarez-Linera J., Iturria-Medina Y., Santarnecchi E., Colom R. (2021). Brain resilience across the general cognitive ability distribution: evidence from structural connectivity. Brain Struct. Funct..

