# Supplementary material for: Network impact score is an independent predictor of post-stroke cognitive impairment: A multicenter cohort study in 2341 patients with acute ischemic stroke
Source: Neuroimage Clin. 2022 Apr 27;34:103018. doi: 10.1016/j.nicl.2022.103018 (PMC9079101; doi:10.1016/j.nicl.2022.103018)
Supplement: Supplementary data 1 [file mmc1.docx]

**Appendix**

**Definition of stroke subtypes.**

Definitions for stroke subtypes were previously described elsewhere.^1^ In short, small subcortical infarcts were defined as single supratentorial infarct without cortical involvement, with a lesion volume of ≤4.19 ml (i.e. a sphere of ≤2 cm diameter; following the STRIVE criteria).^2^ Large subcortical infarcts were defined as supratentorial infarct(s) without cortical involvement, with a lesion volume of >4.19 ml. Cortical infarcts were defined as supratentorial infarct(s) of any volume with cortical involvement.

**Figure A.1. Illustration of the procedure for calculating the network impact score**

**
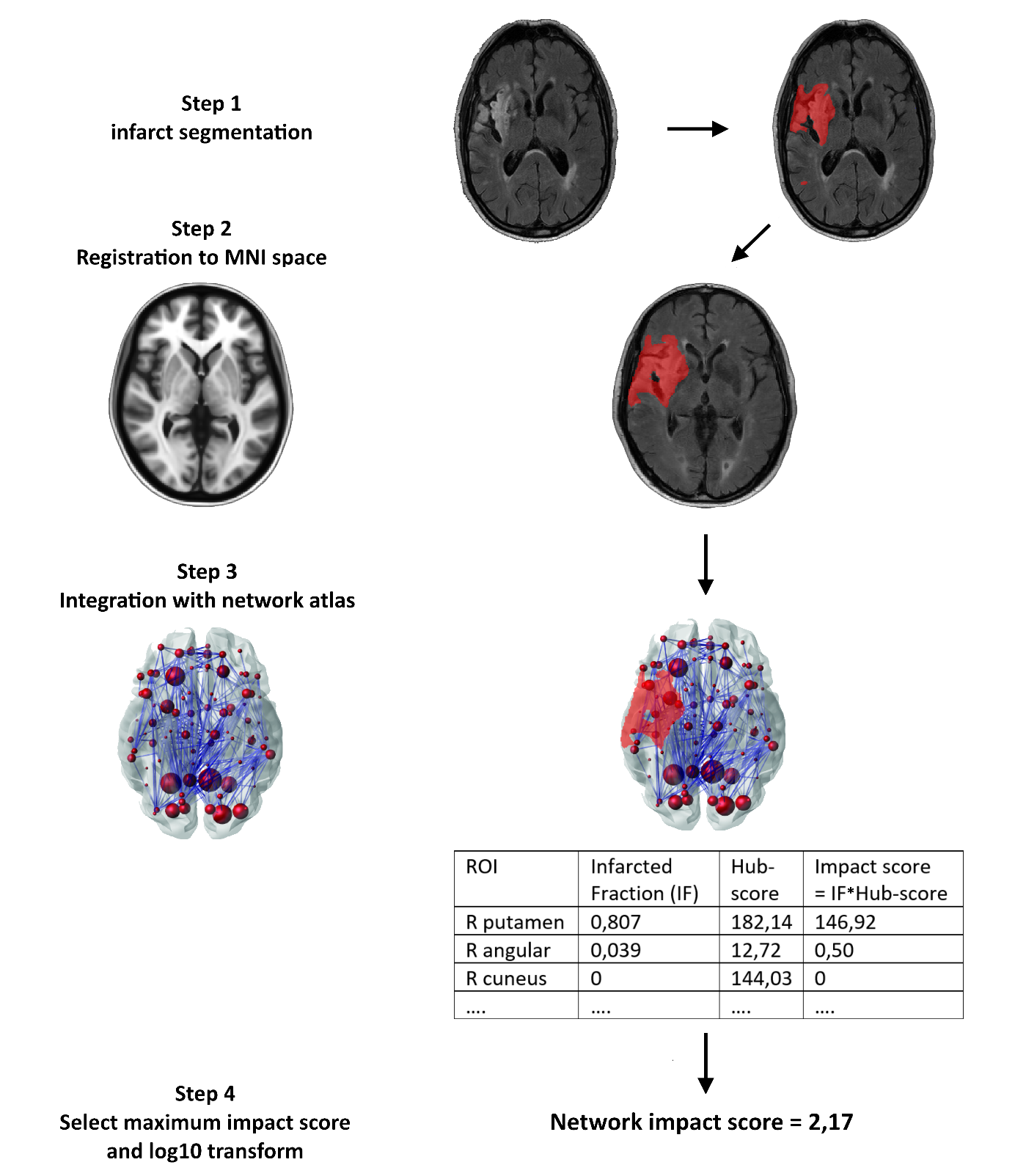
**

**Figure A.2. Boxplots of network impact score for post-stroke cognitive impairment, cognitive recovery and cognitive decline.**


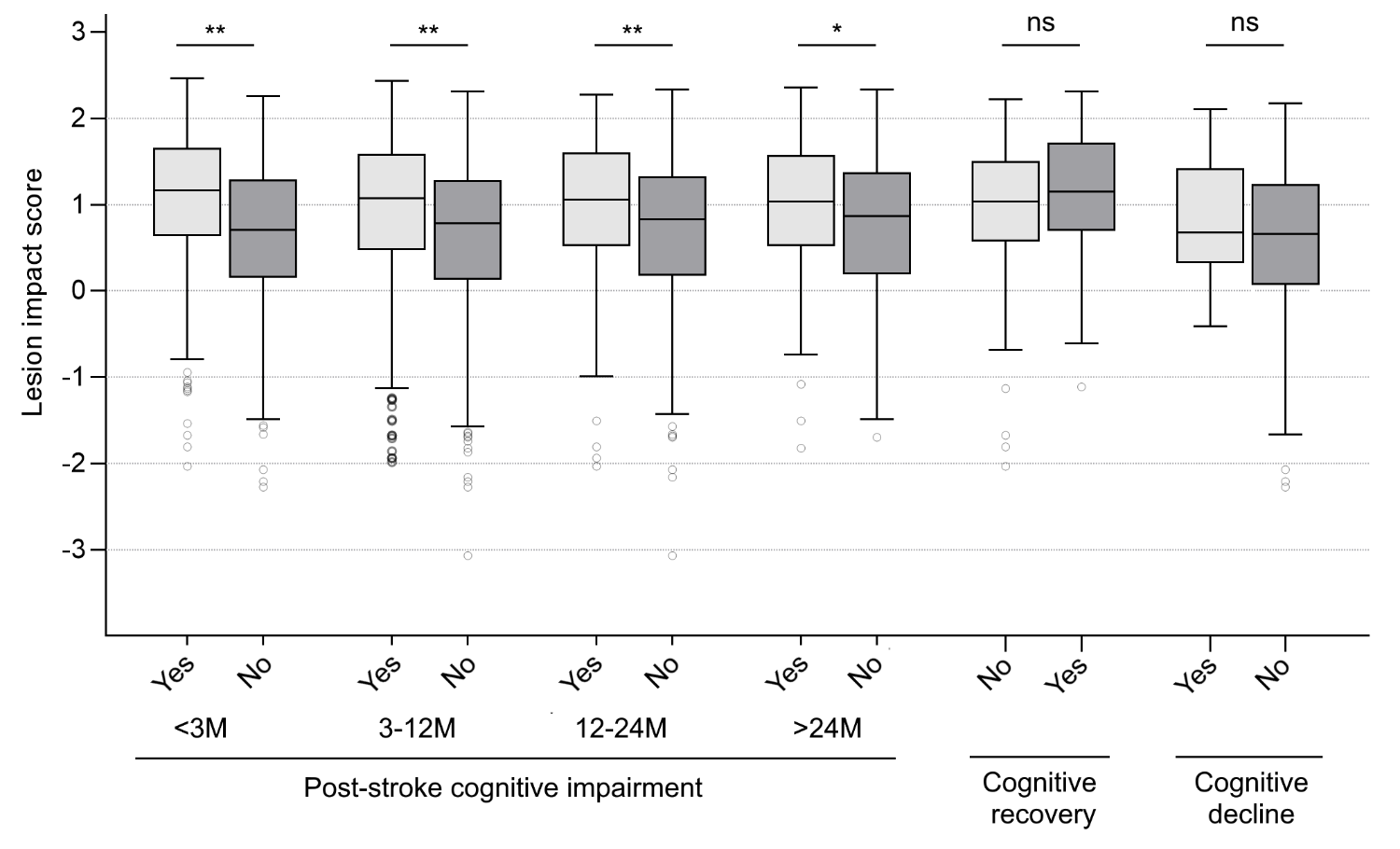


*Boxplots show median and interquartile range; whiskers are maximized at 1.5 times the interquartile range; data points outside the range of the whiskers shown as circles. The numbers of patients per group are provided in Figure 1. Group comparisons refer to the univariable analyses reported in Tables A.3 and A.6.*p<0.01 **p<0.001. NS: not significant.*

**
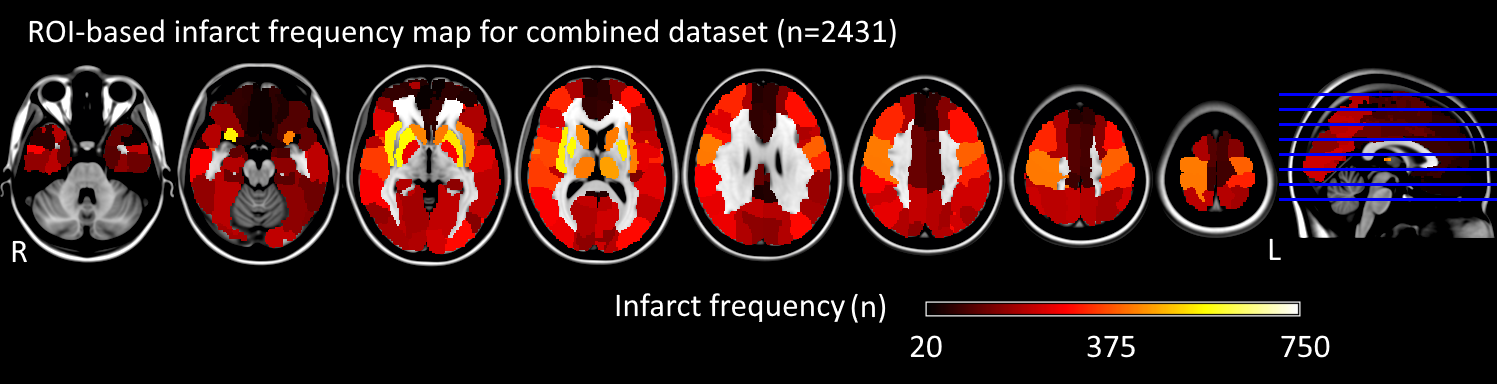
Figure A.3. Infarct prevalence map.**

This figure shows the number of patients with an infarct for each of the 90 regions that are included in the network impact score atlas. Note that ROIs in the vascular territory of the middle cerebral artery, and the basal ganglia and thalamus are most often affected, whereas ROIs in the territory of the anterior cerebral artery are least often affected by infarcts. ROI: region of interest. R: right. L: left. The right hemisphere is depicted on the left.

**Table A.1. Resolution of the brain images on which infarct segmentations were performed.**

| Cohort | Brain image on which segmentation was performed | Resolution of segmented brain image |
| --- | --- | --- |
| Bundang VCI^3^ | DWI or FLAIR | DWI: .86 x .86 x 6 mm  FLAIR: .43 x .43 x 6 mm |
| COAST | DWI, T2 or CT | DWI: 2.4 x 2.4 x 7.0 or 1.25 x 1.25 x 7.0 mm  T2: 0.43 x 0.43 x 7.0 mm  CT: 0.41 x 0.41 x 5.0 mm |
| CODECS | DWI, FLAIR or CT | DWI: 1.0 x 1.0 x 3.0 mm  FLAIR: 0.60 x 0.55 x 0.55 or 0.45 x 0.45 x 5.50 mm  CT: 0.3 x 0.3 x 2.0 or 0.4 x 0.4 x 2.0 mm |
| Hallym VCI^4^ | DWI or FLAIR | DWI: .86 x .86 x 6.5 mm  FLAIR: .43 x .43 x 6.5 mm |
| PROCRAS | FLAIR | 0.45 x 0.45 x 5.0 mm |
| USCOG^5^ | FLAIR or CT | Voxel size along the z-axis (i.e. slice thickness) ranged from 4–6 mm; the in-plane voxelsize (i.e., along x- and y-axis) was less than 1 mm in all cases. |
| CASPER^6^ | FLAIR | .6 x 1.1 x 1.1 mm |
| CROMIS-2 | DWI | 1.25 x 1.25 x 6.50 or 1.2 x 1.2 x 5.0 or  0.94 x 0.94 x 6.0 mm |
| CU-STRIDE | DWI or CT | DWI: 0.90 x 0.90 x 5.5 or 1.80 x 1.80 x 6.5 mm  CT: 0.43 x 0.43 x 5.0 mm |
| GRECogVASC^7^ | 3DT1 | 1.0 x 1.0 x 1.0 mm |
| Mild Stroke Study 2 ^8^ | FLAIR | FoV 24 x 24 cm, matrix 384 (AP) x 224, slice thickness 5 mm (gap 1.0 mm) |
| STROKDEM | DWI | 2.0 x 2.0 x 2.0 mm |

**Table A.2.** Baseline characteristics of the included 2341 patients stratified per cohort.

|  | **Bundang (n=607)** | **CASPER (n=86)** | **COAST (n=65)** | **CODECS (n=0)^b^** | **CROMIS-2**  **(n=88)** | **CU-STRIDE (n=313)** | **GRECogVASC (n=226)** | **Hallym**  **(n=500)** | **MSS-2**  **(n=87)** | **PROCRAS**  **(n=148)** | **STROKDEM (n=121)** | **USCOG**  **(n=100)** |
| --- | --- | --- | --- | --- | --- | --- | --- | --- | --- | --- | --- | --- |
| **Demographics** |  |  |  |  |  |  |  |  |  |  |  |  |
| Age in years, mean (SD) | 69.2 (11.2) | 63.6 (10.6) | 58.3 (9.8) | N/A | 74.0 (9.0) | 68.6 (10.5) | 64.3 (10.5) | 65.0 (12.3) | 66.3 (10.8) | 69.5 (9.4) | 64.7 (12.0) | 59.8 (15.4) |
| Female, n (%) | 249 (41.0) | 25 (29.1) | 18 (27.7) | N/A | 40 (45.5) | 126 (40.3) | 91 (40.3) | 216 (43.2) | 33 (37.9) | 52 (35.1) | 48 (39.7) | 44 (44.0) |
| Education category,^a^ n (%)  - Less than high school  - High school  - Technical/college  - University or higher | 311 (51.2)  129 (21.3)  32 (5.3)  135 (22.2) | 40 (46.5)  13 (15.1)  27 (31.4)  6 (7.0) | 45 (69.2)  12 (18.5)  7 (10.8)  1 (1.5) | N/A | 6 (6.8)  70 (79.5)  N/A  12 (13.6) | 287 (91.7)  5 (1.6)  6 (1.9)  15 (4.8) | 167 (73.9)  21 (9.3)  21 (9.3)  17 (7.5) | 276 (55.2)  120 (24.0)  25 (5.0)  79 (15.8) | 16 (18.4)  55 (63.2)  N/A  16 (18.4) | 63 (42.6)  47 (31.8)  34 (23.0)  4 (2.7) | 77 (63.6)  14 (11.6)  8 (6.6)  22 (18.2) | 40 (40.0)  21 (21.0)  24 (24.0)  15 (15.0) |
| Ethnicity | Korean | Caucasian ** | Singaporean Chinese (70.8%), Malay (21.5%), Indian (7.7%) | N/A | Caucasian** | Chinese | Caucasian | Korean | Caucasian | Caucasian | Caucasian | Caucasian |
| **Clinical characteristics** |  |  |  |  |  |  |  |  |  |  |  |  |
| NIHSS baseline, median (IQR) | 3 (2-6) | N/A | 3 (2-8) | N/A | 3 (2-6)*** | 4 (2-6)* | 3 (2-8) | 2 (1-4)** | 1 (0-2) | 3 (2-5) | 0 (0-1) * | N/A |
| IQCODE, median (IQR) | 3.3 (3.1-3.8)** | 3.1 (3.0-3.3)** | 3.0 (3.0-3.1)** | N/A | 3.0 (3.0-3.3) | N/A | 3.0 (3.0-3.1)** | 3.1 (3.0-3.3)*** | N/A | 3.0 (3.0-3.1)** | 3.0 (3.0-3.1) | N/A |
| Number of cognitive assessments, n (%)  - One  - Two  - Three  - Four  - Five  - Six | 262 (43.2)  148 (24.4)  93 (15.3)  56 (9.2)  19 (3.1)  29 (4.8) | 4 (4.7)  9 (10.5)  37 (43.0)  36 (41.9)  0 (0)  0 (0) | 11 (16.9)  54 (83.1)  0 (0)  0 (0)  0 (0)  0 (0) | N/A | 1 (1.1)  87 (98.9)  0 (0)  0 (0)  0 (0)  0 (0) | 46 (14.7)  38 (12.1)  30 (9.6)  22 (7.0)  75 (24.0)  102 (32.6) | 226 (100)  0 (0)  0 (0)  0 (0)  0 (0)  0 (0) | 481 (96.2)  17 (3.4)  2 (0.4)  0 (0)  0 (0)  0 (0) | 48 (55.2)  39 (44.8)  0 (0)  0 (0)  0 (0)  0 (0) | 9 (6.1)  139 (93.9)  0 (0)  0 (0)  0 (0)  0 (0) | 33 (27.3)  88 (72.7)  0 (0)  0 (0)  0 (0)  0 (0) | 100 (100)  0 (0)  0 (0)  0 (0)  0 (0)  0 (0) |
| Clinical history of stroke, n (%) | 86 (14.2) | 4 (4.7) | 10 (15.4) | N/A | 5 (5.7)* | 36 (11.5) | 16 (7.1) | 67 (13.5)* | 9 (10.3) | 20 (13.5) | 11 (9.1) | 0 (0) |
| **Brain imaging** |  |  |  |  |  |  |  |  |  |  |  |  |
| Scan sequence/modality used for infarct segmentation, n (%)  - DWI  - T2/FLAIR  - CT  - T1 | 590 (97.2)  17 (2.8)  0 (0)  0 (0) | 0 (0)  86 (100)  0 (0)  0 (0) | 25 (38.5)  4 (6.2)  36 (55.4)  0 (0) | N/A | 88 (100)  0 (0)  0 (0)  0 (0) | 223 (71.2)  0 (0)  90 (28.8)  0 (0) | 0 (0)  0 (0)  0 (0)  226 (100) | 487 (97.4)  13 (2.6)  0 (0)  0 (0) | 0 (0)  87 (100)  0 (0)  0 (0) | 0 (0)  148 (100)  0 (0)  0 (0) | 121 (100)  0 (0)  0 (0)  0 (0) | 0 (0)  34 (34.0)  66 (66.0)  0 (0) |
| Normalized acute infarct volume in ml, median (IQR) | 5.6 (1.5-21.9) | 4.2 (1.0-13.6) | 6.7 (1.6-35.1) | N/A | 4.2 (1.5-16.2) | 3.2 (1.2-14.1) | 1.8 (0.4-9.0) | 2.7 (1.0-12.6) | 2.6 (1.2-11.8) | 5.3 (1.8-21.8) | 1.8 (0.8-9.6) | 21.2 (4.0-55.5) |
| Imaging timing, days after event, median (IQR) ^f^ | 5 (4-6) * | 86 (80-99) | 2 (1-4) | N/A | 5 (2-9) | 1 (0-2) | 178 (161-186) | 1 (1-2) | 4 (2-8) | 34 (27-41) | 3 (3-4) | 4 (3-6) |

^a^ Education categories defined by the STROKOG consortium (reference provided in main text). ^b^All patients from the CODECS cohort had isolated infratentorial stroke and were therefore excluded from the current study. * Missing <1%. ** Missing 1-10%. *** Missing >10%. Abbreviations: CT, computed tomography; DWI, diffusion-weighted imaging; FLAIR, fluid attenuated inversion recovery; IQR, interquartile range; SD, standard deviation.

**Table A.3. Association between network impact score and PSCI at stratified timepoints.**

|  | **PSCI <3 months**  398/844 patients (47%) | | **PSCI 3-12 months**  709/1640 (43%) | | **PSCI 12-24 months**  243/853 (28%) | | **PSCI >24 months**  208/522 (40%) | |
| --- | --- | --- | --- | --- | --- | --- | --- | --- |
|  | **OR (95%CI)** | **p-value** | **OR (95%CI)** | **p-value** | **OR (95%CI)** | **p-value** | **OR (95%CI)** | **p-value** |
| **Univariable model** |  |  |  |  |  |  |  |  |
| Network impact score | 177 (1.49-2.11) | <0.001 | 1.51 (1.34-1.70) | <0.001 | 1.46 (1.21-1.77) | <0.001 | 1.43 (1.14-1.80) | 0.002 |
| **Multivariable model ^a^** |  |  |  |  |  |  |  |  |
| Network impact score | 1.34 (1.07-1.69) | 0.012 | 1.33 (1.15-1.55) | <0.001 | 1.24 (0.98-1.58) | 0.076 | 1.31 (0.99-1.74) | 0.063 |

ORs apply to each 1-point increase in the network impact score. Sample sizes in the multivariable models were: 841 (<3 months), 1638 (3-12 months), 852 (12-24 months), and 522 (>24 months) due to exclusion of patients with missing data on clinical history of stroke. ^a^Corrected for age, sex, education, prior stroke, total infarct volume, and study site.

**Table A.4**. Sensitivity analysis after exclusion of patients from the center (Elisabeth Tweesteden ziekenhuis Tilburg, the Netherlands) in which the lesion impact score was originally developed.

|  | **Post-stroke cognitive impairment** | |
| --- | --- | --- |
| **Univariable model** | **OR (95%CI)** | **p-value** |
| Lesion impact score | 1.51 (1.34-1.70) | <0.001 |
| **Multivariable model ^a^** |  |  |
| Lesion impact score | 1.26 (1.09-1.46) | 0.002 |

GEE repeated measures model. ^a^ Corrected for age, sex, education, prior stroke, total infarct volume, and study site. The univariable model includes 4370 cognitive assessments in 2193 patients. The multivariable model includes 4364 cognitive assessments in 2188 patients (five patients were excluded in the multivariable model due to missing data on clinical history of stroke). The OR applies to each 1-point increase in the lesion impact score.

**Table A.5**. Sensitivity analysis after exclusion of patients from the center (Elisabeth Tweesteden ziekenhuis Tilburg, the Netherlands) in which the lesion impact score was originally developed.

|  | **PSCI <3 months** | | **PSCI 3-12 months** | | **PSCI 12-24 months** | | **PSCI >24 months** | |
| --- | --- | --- | --- | --- | --- | --- | --- | --- |
|  | **OR (95% CI)** | **p-value** | **OR (95% CI)** | **p-value** | **OR (95% CI)** | **p-value** | **OR (95% CI)** | **p-value** |
| **Univariable model** |  |  |  |  |  |  |  |  |
| Lesion impact score | 1.87 (1.53-2.28) | <0.001 | 1.55 (1.37-1.76) | <0.001 | 1.43 (1.17-1.74) | <0.001 | 1.43 (1.14-1.80) | 0.002 |
| **Multivariable model ^a^** |  |  |  |  |  |  |  |  |
| Lesion impact score | 1.31 (1.01-1.71) | 0.044 | 1.36 (1.17-1.59) | <0.001 | 1.19 (0.92-1.53) | 0.182 | 1.31 (0.99-1.74) | 0.063 |

Logistic regression models for post-stroke cognitive impairment at specific timepoints. ^a^ Corrected for age, sex, education, prior stroke, total infarct volume, and study site. The ORs apply to each 1-point increase in the lesion impact score. Sample sizes in respectively the univariable and multivariable models were: 696 and 693 (<3 months), 1568 and 1566 (3-12 months), 786 and 785 (12-24 months), 522 and 522 (>24 months). The lower numbers in multivariable models are due to exclusion of patients with missing data on clinical history of stroke.

**Table A.6**. Association between the network impact score and cognitive recovery and decline.

|  | **Cognitive recovery**  64/181 patients (35%) | | **Cognitive decline**  26/287 patients (9%) | |
| --- | --- | --- | --- | --- |
|  | **OR (95% CI)** | **p-value** | **OR (95% CI)** | **p-value** |
| **Univariable model** |  |  |  |  |
| Lesion impact score | 1.31 (0.88-1.93) | 0.184 | 1.49 (0.90-2.46) | 0.124 |
| **Multivariable model ^a^** |  |  |  |  |
| Lesion impact score | 1.38 (0.83-2.29) | 0.212 | 1.36 (0.70-2.63) | 0.363 |

Logistic regression models for post-stroke cognitive recovery and decline. ^a^ Corrected for age, sex, education, prior stroke, total infarct volume, and study site. The ORs apply to each 1-point increase in the lesion impact score. Sample sizes in respectively the univariable and multivariable models were: 181 and 180 (cognitive recovery), 287 and 287 (cognitive decline). The lower numbers in the multivariable model for cognitive recovery is due to exclusion of a patient with missing data on clinical history of stroke.

**Table A.7**. Association between the network impact score and cognitive recovery and decline: Sensitivity analysis after exclusion of patients from the center (Elisabeth Tweesteden ziekenhuis Tilburg, the Netherlands) in which the lesion impact score was originally developed.

|  | **Cognitive recovery**  42/132 patients (32%) | | **Cognitive decline**  18/197 patients (9%) | |
| --- | --- | --- | --- | --- |
|  | **OR (95% CI)** | **p-value** | **OR (95% CI)** | **p-value** |
| **Univariable model** |  |  |  |  |
| Lesion impact score | 1.24 (0.77-1.98) | 0.379 | 1.24 (0.68-2.26) | 0.477 |
| **Multivariable model ^a^** |  |  |  |  |
| Lesion impact score | 1.34 (0.69-2.57) | 0.388 | 1.46 (0.60-3.54) | 0.402 |

Logistic regression models for post-stroke cognitive recovery and decline. ^a^ Corrected for age, sex, education, prior stroke, total infarct volume, and study site. The ORs apply to each 1-point increase in the lesion impact score. Sample sizes in respectively the univariable and multivariable models were: 132 and 131 (cognitive recovery), 197 and 197 (cognitive decline). The lower number in the multivariable model for cognitive recovery is due to exclusion of a patient with missing data on clinical history of stroke.

**References**

1. Weaver NA, Kuijf HJ, Aben HP, et al. Strategic infarct locations for post-stroke cognitive impairment: a pooled analysis of individual patient data from 12 acute ischaemic stroke cohorts. *Lancet Neurol* 2021; 20: 448–459.

2. Wardlaw JM, Smith EE, Biessels GJ, et al. Neuroimaging standards for research into small vessel disease and its contribution to ageing and neurodegeneration. *The Lancet Neurology* 2013; 12: 822–838.

3. Biesbroek JM, Lim J-S, Weaver NA, et al. Anatomy of phonemic and semantic fluency: A lesion and disconnectome study in 1231 stroke patients. *Cortex* 2021; 143: 148–163.

4. Biesbroek JM, van Zandvoort MJE, Kappelle LJ, et al. Shared and distinct anatomical correlates of semantic and phonemic fluency revealed by lesion-symptom mapping in patients with ischemic stroke. *Brain Struct Funct* 2016; 221: 2123–2134.

5. Biesbroek JM, van Zandvoort MJE, Kappelle LJ, et al. Distinct anatomical correlates of discriminability and criterion setting in verbal recognition memory revealed by lesion-symptom mapping. *Hum Brain Mapp* 2015; 36: 1292–1303.

6. Douven E, Schievink SHJ, Verhey FRJ, et al. The Cognition and Affect after Stroke - a Prospective Evaluation of Risks (CASPER) study: Rationale and design. *BMC Neurol* 2016; 16: 65.

7. Puy L, Barbay M, Roussel M, et al. Neuroimaging determinants of Poststroke cognitive performance: The GRECogVASC Study. *Stroke* 2018; 49: 2666–2673.

8. Wardlaw JM, Makin SJ, Valdés Hernández MC, et al. Blood-brain barrier failure as a core mechanism in cerebral small vessel disease and dementia: evidence from a cohort study. *Alzheimer’s Dement* 2017; 13: 634–643.
